# Psychological repercussions of breast or uterine cervical cancer disclosure to women in Gabon

**DOI:** 10.1371/journal.pone.0326378

**Published:** 2025-06-20

**Authors:** Béa-Christelle Ndjengue Bengone, Jean Toniolo, Angela-Christie Filankembo Kava, Edgard Brice Ngoungou, Ernest Belembaogo, Pierre-Marie Preux

**Affiliations:** 1 Inserm U1094, IRD UMR270, Univ. Limoges, CHU Limoges, EpiMaCT - Epidemiology of Chronic Diseases in Tropical Zone, Institute of Epidemiology and Tropical Neurology, Omega Health, Limoges, France; 2 Research Unit of Chronic Diseases and Environmental Health (UREMCSE), University of Health Sciences, Libreville, Gabon; 3 Institut de Cancérologie d’Akanda (ICA), Akanda, Gabon; 4 University Department of Nursing Sciences, Faculty of Medecine, University of Limoges, France; 5 Department of Epidemiology, Biostatistics and Medical Informatics (DEBIM). University of Health Sciences, Libreville, Gabon; Freelance Medical Research and Writing, UNITED KINGDOM OF GREAT BRITAIN AND NORTHERN IRELAND

## Abstract

**Background:**

Breast and cervical cancers are major health issues globally, with particularly high burdens in sub-Saharan Africa, including Gabon. Limited epidemiological data and insufficient attention to the psychological impacts of these cancers highlight critical research gaps. This study aimed to explore the psychological repercussions experienced by Gabonese women following the disclosure of a breast or cervical cancer diagnosis.

**Methods:**

A mixed-methods cross-sectional study was conducted from March to May 2023 at the Institut de Cancérologie d’Akanda. Women recently diagnosed with breast or cervical cancer were recruited using a consecutive sampling method. Data were collected through semi-structured individual interviews and one focus group to explore emotional responses, coping strategies, and perceptions of patient–provider interactions. In addition, the Patient Health Questionnaire-9 (PHQ-9) was administered to assess depressive symptoms.

**Results:**

Thirteen Gabonese women diagnosed with breast (n = 10) or cervical cancer (n = 3) participated in this mixed-methods study. Following the disclosure, all patients reported depressive symptoms ranging from minimal to moderately severe (PHQ-9). Qualitative analysis revealed intense emotional distress, including anxiety, sadness, sleep disturbances, and despair. The diagnosis affected participants’ identity, body image, and social relationships, reinforcing feelings of stigma, isolation, and loss. Beliefs about disease etiology and social representations shaped emotional responses. While some relied on spirituality, social support, or personal resilience to cope, others expressed unmet psychological needs. Participants emphasized the importance of clear, compassionate communication from healthcare professionals, highlighting the need for empathy, trust, and active listening during diagnosis disclosure.

**Conclusion:**

This pioneering study in Gabon identifies profound psychological effects associated with breast and cervical cancer diagnosis disclosure. The findings underscore the urgent need for improved psychosocial support, effective communication training for healthcare providers, and culturally tailored interventions to address mental health concerns in this population.

## Introduction

Breast cancer is the most frequently diagnosed malignancy in women globally [1,2]. The World Health Organization (WHO) reported approximately 2.3 million new cases in 2020, representing about 11.7% of all new cancer diagnoses [3]. Cervical cancer ranks as the fourth most prevalent malignancy among women, with around 604,000 new cases identified in 2020 [4]. The incidence of cervical cancer is notably higher in low- and middle-income countries [1,3,5,6]. Breast and cervical cancers account for more than half of the cancer burden among women in sub- Saharan Africa. It is also the leading cause of gynecological cancer-related deaths worldwide [1,4]. In 2022, the estimated mortality rates of breast and cervical cancer in Africa were 21.9 per 100.000 women and 19.3 per 100.000 women, respectively [7]. This high mortality may be partly attributed to diagnostic delays, as late detection significantly reduces the chances of survival and limits timely access to effective treatment. Delays in cancer diagnosis are multifactorial, encompassing inadequate screening programs, limited awareness of symptoms and risk factors, insufficient attention to women’s health concerns, restricted access to healthcare facilities, and the influence of sociocultural beliefs and familial dynamics [8–10]. According to data projections, only one in two women diagnosed with cancer has the chance of still being alive five years after the cancer diagnosis [11].

Based on Global Cancer Observatory (Globocan) data, it was predicted that by 2022, there would be 1875 new cases of cancer per 100,000 inhabitants among the 2.3 million people living in Gabon [1]. During the same period, the prevalence of cancers in women in Gabon would be 1133 per 100,000 inhabitants and that of breast and cervical cancers combined would be 594 per 100,000 women; with mortality rates of 614 per 100,000 for all-cause cancers, 113 per 100,000 women for breast cancer and 139 per 100,000 women for cervical cancer [4]. Although a retrospective study conducted at the Institut de Cancer d’Akanda (ICA) by Ivanga et al between 2013–2017 revealed a deficit in epidemiological data on cancers in Gabon [12], the GLOBOCAN estimates underscore an urgent need for comprehensive cancer research in Gabon to guide the implementation of appropriate prevention and management interventions [12]. From a socio-cultural perspective, the perception of cancer among women in several African countries is commonly associated with suffering, bodily disfigurement, fear, and mortality [13–16]. However, in Gabon, no study to date has specifically documented this representation. These issues, coupled with the high burden of breast and cervical cancers in women in Gabon, highlight a need to also consider the psychological impact of cancer diagnosis in research and management frameworks of breast and cervical cancers in Gabon. In this context, the present study aimed to describe the psychological impact of the diagnosis disclosure to Gabonese women diagnosed with breast or cervical cancer. Specifically, it seeked to identify the main psychological challenges encountered, and explore the patients’ needs to overcome those challenges

## Methods

### Study design & patients

A cross-sectional mixed-methods study [17,18] was conducted at the ICA between March and May 2023. The study was reported in accordance with the COREQ guidelines (appendix 1) for the qualitative component and the STROBE guidelines (appendix 2) for the quantitative component.

Patients were identified consecutively based on the reception office registry and manual patient records. A non-probability consecutive sampling approach was employed in this study. Participants were required to be aged 18 years or older and to have received a recent diagnosis of breast or cervical cancer [18,19]. The study excluded patients with cognitive deficits or those unable to communicate. We anticipated including a minimum of nine participants with the possibility of increasing this number to approach data saturation [20]. All patients identified between March and May 2023 were contacted by phone by second-year master student’s in clinical psychology, who had received prior training and were responsible for presenting the study and its objectives to the patients. When these patients expressed a positive interest or desire to learn more before giving a definitive agreement, an appointment was scheduled with a certified researcher in conducting interviews (BCN) to participate in a semi-structured interview as part of the qualitative research phase. It was also possible for participants to engage in a focus group. Prior to inclusion in the study, written informed consent was obtained from all participants. The principles of confidentiality and anonymity were scrupulously observed.

### Study setting

Gabon’s healthcare system is organized into a hierarchical structure, with University Hospital Centers and specialized institutional facilities at the top. The ICA, located in northern Greater Libreville, is the country’s leading cancer center. Established in 2012, the ICA has recorded nearly 6,000 cancer cases to date [21]. In 2022, 530 new cancer cases were recorded, including 111 breast cancer cases and 49 cases of cervical cancer [21]. Newly referred patients, accompanied by diagnostic test results and referral letters, are registered at the ICA and scheduled for consultations with medical or radiation oncologists.

### Data collection

To ensure the impartiality of the data collection process, measures were implemented to guaranteed that none of the patients had any prior relationship with the member of the research team conducting the interviews.

#### Qualitative data collection.

A semi-structured interview guide was developed, allowing flexibility while maintaining a focus on key research areas. The guide consisted of open-ended questions designed to elicit detailed narratives regarding patients’ experiences regarding the disclosure of cancer diagnosis and its associated impacts. These questions were informed by a comprehensive review of existing literature [14,22] and specific thematic considerations relevant to this study. Themes explored included the patient’s journey from initial symptom identification to definitive diagnosis, intricacies of the diagnosis disclosure process, the nature and extent of information provided, and the multifaceted effects of the illness on familial, social, and professional domains. Additionally, psychological dimensions were examined through questions addressing pre- and post-diagnosis perceptions of the illness, methods employed by healthcare professionals during the disclosure of the diagnosis, immediate patients’ emotional responses, and patients’ coping strategies.

Face-to-face individual interviews were conducted privately to foster their trust and encourage open responses. Researchers maintained reflexive diaries throughout the interview process to document observations, identify potential patients’ biases, and record insights, thus enhancing transparency and rigor in the research methodology.

Among the thirteen patients enrolled in the study, seven participated in a subsequent focus group. The group was purposefully heterogeneous to capture a diversity of perspectives and to elicit shared experiences and collective insights. It served to complement individual interviews by enabling interactive discussion and the identification of common themes. The session lasted approximately 90 minutes and was facilitated by an experienced moderator who ensured equitable participation. Discussions focused on the context of cancer detection and disclosure, key factors identified by patients as critical to effective patient-clinician interactions, and the preferred attributes of the individual responsible for delivering the cancer diagnosis. Focus group data were analyzed alongside individual interview data to identify convergent and divergent themes, thereby enhancing the depth of analysis.

All semi-structured interviews and the focus group were audio-recorded in full to ensure accurate transcription and support rigorous analysis.

#### Quantitative data collection.

Quantitative data were collected using a questionnaire, including sociodemographic data (age, educational level, marital status, occupation, religion, residence, and number of children), clinical data (tumor location, symptom onset, cancer, stage at diagnosis, family history of cancer, date of first medical consultation, comorbidities, and cancer treatment types) and the individuals responsible for disclosing the cancer diagnosis.

Following the interview, patients completed the Patient Health Questionnaire-9 (PHQ-9), a validated self-report measure assessing depression symptoms over the last two weeks and depression severity based on nine core criteria of the 4th edition of the Diagnostic and Statistical Manual of Mental Disorders. Each of the nine mandatory questions of the PHQ-9 questionnaire (S3 Appendix) are rated from 0 to 3, with 0 corresponding to the response “never” and 3 corresponding to the response “nearly every day”. Accordingly, total PHQ-9 scores range from 0 to 27. The PHQ-9 questionnaire also has an additional question which can be asked to patients to assess the influence of depressive symptoms on their daily functioning [23,24]. It is important to note that the questionnaire also demonstrated a good reliability and validity for detecting major depressive episodes in sub-Saharan Africans, including those with cancers [25,26].

### Data analysis

Qualitative data were analyzed using reflexive thematic analysis, while quantitative data were examined through descriptive statistical procedures.

Descriptive statistical analyses were performed using SPSS® version 25, with preliminary data organization in Excel. Due to the small sample size, variables are primarily presented as absolute frequencies. The PHQ-9 was scored according to the guidelines established by its original authors [24,27]. This standardized interpretation facilitated the classification of depressive symptom severity among participants. Scores are categorized as follows: 0–4 indicates minimal depression, with little or no clinically significant symptoms; 5–9 corresponds to mild depression, with limited impact on daily functioning; 10–14 reflects moderate depression, likely to interfere with routine activities and well-being; 15–19 denotes moderately severe depression, associated with marked impairment in personal, social, or occupational domains; and 20–27 indicates severe depression, characterized by significant functional impairment and psychological distress [24].

Participants completed the PHQ-9 independently to ensure anonymity and reduce interviewer bias. For missing data, a score of 0 was assigned when items were unanswered. If less than 20% of items (≤2/9) were missing for a participant, the mean of completed items was imputed [27]. To assess the prevalence of depressive symptoms, total and item-level scores were analyzed to identify the most frequently elevated symptoms across the sample.

We performed a topic summary-based thematic analysis which consisted of structuring data from the semi-structured interviews and focus group discussions into clear thematic categories that summarize patients’ experiences in a descriptive manner. All interviews and focus group discussions were transcribed verbatim, capturing verbal details such as pauses and hesitations. The analysis began with an in-depth immersion in the data through repeated readings of the transcripts to grasp the nuances of participants’ narratives. Preliminary notes and memos were documented to identify emerging ideas and recurrent patterns, forming the basis for subsequent coding.

Researchers (BCN and JT) independently conducted initial coding and thematic identification, ensuring analytical triangulation and reducing bias. NVivo 11® software facilitated data management, systematic coding, and category development, enhancing the transparency and reliability of the analysis [28].

Although the concept of data saturation remains debated in qualitative research [19], we adhered to established methodological guidelines [29,30], to approach it pragmatically. Data collection continued until thematic saturation was reached—that is, when no new themes or insights emerged from successive interviews. This point was typically reached after approximately nine interviews, consistent with published benchmarks on saturation in qualitative research [18,31].

A reflexive thematic analysis was conducted on all transcripts, enabling a comprehensive exploration of participants’ experiences. To ensure rigor and reflexivity, three structured meetings were held with the research team (BCN, JT, AFK) at key stages of the analysis—initial, midpoint, and final each lasting approximately one hour. These sessions allowed for collaborative review, theme refinement, and interpretive depth.

To maintain their confidentiality, patients were identified using PxS and PxC codes for participants involved in semi-structured interviews and focus group discussions, respectively, where x is the number in ascending order of participation in the semi-structured interview or focus group discussion

### Ethical considerations

The study was approved by the Director of the Institut de Cancérologie d’Akanda and its Evaluation Committe (Approval No. 1-AP-26/2023.MDN/DGSSM/ICA/ONCO-MED). All data were securely stored on a password-protected computer, accessible only to the research team. The study was conducted in compliance with the Declaration of Helsinki- 2024 [32].

## Results

### Patients’ characteristics

**Fig 1:**
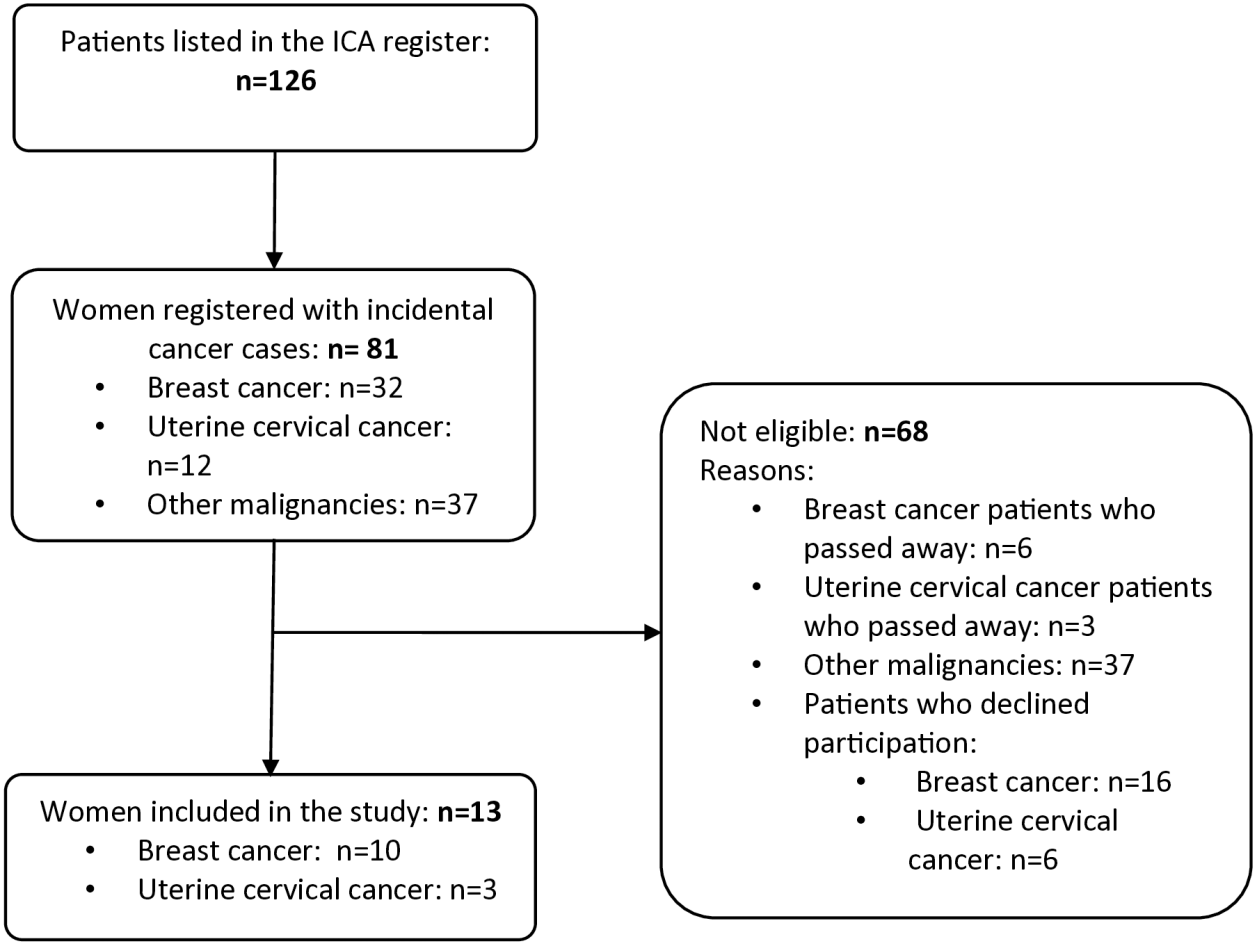
Flow chart of the patient’s selection process.

A total of 13 women were included, comprising 10 with breast cancer and 3 with cervical cancer. Fig 1 illustrates the patient selection procedure, and Table 1 provides a comprehensive overview of the patients’ characteristics.

**Table 1:** Participant Characteristics.

Patient’s identity	Age (years)	Religion	Cancer types	Stage cancers	Conditions of discovery	Living environment	Medical history	Previous or ongoing cancer treatment	Side effects of cancer treatments	Marital status/ number of children	Education level/ socio professional status	Comorbidities at the time of cancer diagnosis
P1S	45	Catholic	Breast	TNM IV	Medical check up	Urban area	NS	No treatment	NA	Single 4	Secondary school Unemployed	Sickle cell disease
P2S	46	Evangelical	Breast	TNM IV	Fortuitous discovery	Urban area	Paternal grandmother/ Maternal aunt: breast cancer Brother: Nasopharyngeal cancer	Chemotherapy	NS	Married 3	Higher education Unemployed	NS
P3S	52	Evangelical	Breast	TNM III	Fortuitous discovery	Urban area	NS	Chemotherapy	Nausea, alopecia, fatigue, vomiting	Married 2	Secondary school Cashier	HTN
P4S	43	Catholic	Breast	TNM II	Medical check up	Urban area	NS	Not treatment	NS	Separated 4	Secondary school Teacher	NS
P5S	41	Evangelical	Breast	TNM I	Fortuitous discovery	Urban area	Previous cesarean section	Lumpectomy, Chemotherapy radiotherapy mastectomy	Nausea, joint pain, vomiting	Single 1	Higher education Administrative Manager	NS
P6S	43	NS	Breast	TNM III	Fortuitous discovery	Urban area	Sisters: breast cancer	Not treatment	NS	Widowed 6	Secondary school Healthcare assistant	Diabetes mellitus, Overweight
P7S	60	Catholic	Breast	TNM IV	Fortuitous discovery	Urban area	NS	Not treatment	NS	Single 1	Higher education Retired	HTN
P8S	55	NS	Breast	TNM IV	Fortuitous discovery	Urban area	NS	Chemotherapy radiotherapy	alopecia, anemia, diarrhea	Single 3	Secondary school Policewoman	NS
P9S	52	Protestant	Breast	TNM III	Fortuitous discovery	Rural area	NS	Not treatment	NA	Common law-union 7	Primary school farmer	HTN
P10S	56	Catholic	Breast	TNM IV	Fortuitous discovery	Rural area	NS	Not treatment	NA	Widowed 6	Secondary school unemployed	NS
P1C	64	Protestant	Cervical uterine	FIGO III	Fortuitous discovery	Rural area	NS	Chemotherapy	Nausea, alopecia, fatigue,	Widowed 4	Secondary school merchant	HTN
P2C	56	Catholic	Cervical uterine	FIGO I	Fortuitous discovery	Urban area	Sisters/ maternal aunt: breast cancer	Hysterectomy chemotherapy radiotherapy	joint pain, alopecia, anemia	Single 2	Secondary school Call center agent	HTN
P3C	42	Catholic	Cervical uterine	FIGO I	Fortuitous discovery	Rural area	NS	Hysterectomy chemotherapy radiotherapy	Diarrhea, joint pain, anemia, fatigue	Common law-Union 3	Secondary school unemployed	NS

“NS”: Not specified.

“NA”: Not Applicable.

“Previous or ongoing cancer treatment” Prior or current anticancer treatment encompasses medical interventions (surgery, chemotherapy, radiotherapy, immunotherapy, hormonotherapy) directed at the malignancy.

“Not treatment”. At the time of assessment, the patients had not yet initiated their therapeutic regimens. This was attributable, in some instances, to ongoing complementary diagnostic investigations, while in others, treatment commencement was scheduled for the immediate future.

“Fortuitous discovery”, within the context of this study, refers to a self-detection of clinical signs by the woman, subsequent to the persistence of symptoms. This mode of discovery is characterized by the absence of a prior medical consultation aimed at establishing a diagnosis of the disease through clinical or paraclinical examinations. Nevertheless, the occurrence of various warning signs, such as an atypical breast mass, symptomatic axillary lymphadenopathy, or intermenstrual or postmenopausal bleeding, prompted the women to consult a healthcare facility or participate in a screening campaign to benefit from medical check-up.

“HTN”: Hypertension or High blood pressure.

“FIGO” (International Federation of Obstetrics and Gynecology) is the international stage classification for cervical cancer https://www.sciencedirect.com/science/article/abs/pii/S1776981721000031 [33], while the American Joint Committee on Cancer’s “TNM “stage classification for breast cancer is an international standard code used by cancer teams to describe the extent of a cancer. T = Tumour, N = Node, M = Metastasis. We referred to information collected from the patients’ physical files https://ishh.fr/cancer-du-sein/stades-du-cancer-du-sein/ [34].

The participants’ ages ranged from 41 to 64 years, with nine having attained a secondary school level of education and four being unemployed. In 11 patients, cancer was discovered fortuitously. Cervical cancer stages ranged from FIGO I to FIGO III [33], while breast cancer stages spanned from TNM I to TNM IV [34].

The average time from initial symptoms presentation and diagnosis disclosure was four months, ranging from 730 days to four years. The average time from diagnosis disclosure and the individual interviews (data collection) was 137 ± 74.3 months. The interviews lasted on average 51 ± 12.3 minutes.

Regarding psychological support following diagnosis, Patient PS6 reported attending two sessions with a psychologist after the diagnosis disclosure but voluntarily discontinued these sessions three months prior to the study. Patient PS1 received the diagnosis disclosure in the presence of both a physician and a psychologist and subsequently benefited from immediate psychological follow-up, which was maintained throughout the study period.

Within the patient cohort, six individuals had not yet commenced treatment as they were still undergoing complementary diagnostic procedures, and seven had been undergoing treatment for more than three months.

### Psychological repercussions of cancer disclosure on patients

#### PHQ-9-based depression scores.

PHQ-9 scores ranged from 2 to 18, with six patients having mild depression and two having minimal depression. The analysis revealed the following item scores: Item 4, Item 3, Item 2, and Item 5 had the most elevated aggregate scores at 20, 18, 15 and 15, respectively, according to the PHQ-9 questionnaire. These items correspond to the core symptomatology of depression commonly associated with disorders, including asthenia, sleep disturbances, altered appetite, and affective disturbances characterized by feelings of despair, sadness, and depression. Likewise, depressive symptoms did not have a repercussion on the daily functioning of eight patients. More details about PHQ-9 assessment of patients is available in Table 2.

**Table 2:** PHQ-9 scores.

Patient’s identity	Item 1		Item 2	Item 3	Item 4	Item 5	Item 6	Item 7	Item 8	Item 9	Total score	Category	Optional item score	Repercussion of depressive symptoms on daily functioning
**P1S**		2	0	2	3	0	0	2	0	0	**9**	Mild depression	0	None
**P2S**			3	3		3	3	3	3	0	**18**	Moderately severe	2	Very high
**P3S**		0	1	3	1	1	3	0	0	1	**10**	Moderate	0	None
**P4S**		0	0	0	1	1	0	0	0	0	**2**	Minimal depression	0	None
**P5S**		1	2	0	1	0	2	1	0	0	**7**	Mild depression	2	Very high
**P6S**		0	2	0	0	1	0	0	3	0	**6**	Mild depression	0	None
**P7S**		3	1	1	3	3	1	3	2	0	**17**	Moderately severe	0	None
**P8S**		1	1	1	3	0	0	0	0	0	**6**	Mild depression	1	Quite high
**P9S**		3	0	1	3	1	0	0	0	0	**8**	Mild depression	0	None
**P10S**		1	0	3	1	2	0	3	0	0	**10**	Moderate depression	0	None
**P1C**		0	0	2	0	0	0	0	0	0	**2**	Minimal depression	1	Quite high
**P2C**		0	3	0	3	0	1	0	2	0	**9**	Mild depression	2	Very high
**P3C**		0	2	2	1	3	0	0	0	0	**8**	Mild depression	0	None
Aggregate score for each mandatory item		**11**	**15**	**18**	**20**	**15**	**10**	**12**	**10**	**1**				

#### Emotional impact.

From reflective thematic analysis, it appeared that the diagnosis of a serious illnesses such as breast and cervical uterine cancers elicits a multitude of complex and often contradictory emotional responses. Patients expressed experiencing profound anxiety and intense fear due to the uncertainty about the future.

*“While people sleep at night, you reflect. How will tomorrow be? How will I be tomorrow?” P2S.*

The confrontation with feelings of imminent death was also a recurring theme, which understandably provoked emotions such as anger and sadness, or denial reactions in some cases. Patients’ accounts reveal the enduring impact of a cancer diagnosis on emotional health, even years after it is disclosed. As one patient expressed:

*“Since December, when I was informed, I cry... I often think, I rethink... (silence) and when I think, I cry... (sobs)” P9S.*

*“It was difficult. It’s still difficult. I can’t say it’s over... it’s difficult; it’s too difficult. We accept it, yes... but the fear is there... the difficulty is there...” P5S.*

These comments highlight the difficulty of fully overcoming the psychological aftermath of the diagnosis disclosure. The memory of this pivotal moment remains vivid, this intense emotional state persists, as another patient said during the focus group:

*“Does time really erase this moment? I don’t think so. You think you’ve forgotten about it, but it resurfaces when you least expect it...” P3S.*

The fear of death was especially pronounced among those who had previously witnessed the demise of loved ones with similar cancers as one patient recounted:

*“I knew of a woman who had breast cancer... she died with one breast... I thought of her and said to myself, she died of breast cancer, so will I die too...” P1S.*

Table 3 summarizes the patients’ specific emotional reactions.

**Table 3:** Emotional responses reported by patients upon cancer diagnosis disclosure.

Theme	Sub-theme	Verbatim	Patient’s identity
Emotions	**Fear of death**	*“They say cancer kills. If you are told that this kills you, what* *are you going to do? You are afraid... I was so afraid...“ “People say that when you have this illness, your years are counted... you cannot live long.”* *“As soon as she told me (the doctor), I saw death, immediately...”* *“For me, cancer is death, as you go along you deteriorate and die...it always makes you think of death...”.* *“I knew of a woman who had breast cancer... she died with one breast... I thought of her and said to myself, she died of breast cancer, so will I die too”.*	*P2S* *P6S* *P5S* *P4S* *P1S*
	**Anxiety**	*“It must be said that it is not easy when this is announced, because right away, you start to think. I had a lot of questions in my head... that were troubling me... in my brain...”*	*P2S*
	**Sadness/ Despair/ Emotional Pain**	*“Just a little sadness, not much...”* *“I felt very bad...”* *“I cried for days.”* *“I was too desperate, I spent my time crying, my mind was elsewhere...”* *“It was too difficult... I couldn’t sleep, I wasn’t eating anymore, I even lost weight…”* *“It hurts me... I cannot hide it... it really hurts...”*	*P1S* *P2S* *P3C* *P9S* *P5S* *P2C*
	**Anger**	*“I was angry at myself because I wondered how did I not see this and how did I not feel anything. I was especially really angry at myself...”* *“I was angry at myself because I wondered how did I not see this and how did I not feel myself... (silence and crying fits)”*	*P1S* *P8S*
	**Guilt**	*“It gives the impression that you have a dirty disease... like a disease you picked up outside...”* *“I blamed myself...”*	*P6S* *P1S*
	**Emotional shock**	*“I felt like I was struck by lightning; I was surprised to hear the word cancer. I was like hypnotized, paralyzed...”* *“I was shocked... very shocked...”* *“I felt an emptiness inside me... it really shook me... for a moment, when she was talking, I was elsewhere, really like an emptiness, I wasn’t thinking about anything, I stayed like that for a while... (silence)”*	*P10S* *P4S* *P1S*
	**Surprise**	*“I was astonished; you ask yourself why I? why... what a* *surprise?“* *“...the element of surprise...”*	*P2S* *P1S*
	**Crying**	*“Upon receiving the news, I cried so much... as if my brain... my thoughts... everything was elsewhere, I was curling up...”* *“I collapsed... I saw, I do not know how to explain it. I really cried, all day long, I cried...”* *“I held back some tears, I didn’t want anyone to see me cry...”* *“I felt very bad and cried. I cried so much...”*	*P9S* *P2C* *P1S* *P2S*

#### Perceptions and representations about cancer.

Patients drew parallels between breast or cervical cancer and other socially stigmatized conditions, such as HIV/AIDS. These comparisons reflected internalized stigma and underscored the psychological burden associated with the diagnosis:

*“Cancer is like a sexually transmitted disease, for example. Worse than HIV...”. P6S,*

*“It’s like before with AIDS. We saw that, we saw death. But now we live with it, we get over it, we have children. It’s the same with cancer.” P4S.*

Such analogies revealed enduring negative perceptions of cancer, portraying it as both socially discrediting and emotionally distressing.

Verbatim transcripts also revealed patient reflections on the etiology of their illness, especially in cases without a known family history. Attributions regarding the causes of breast or cervical cancer shaped how patients processed the disclosure, influencing emotional responses. These beliefs either triggered guilt or modulated anxiety—mitigating or exacerbating distress related to future uncertainty.

*“I’ve never heard of it, even in my family...There are no cases in my family... I don’t know what cancer is… what is it, fat, worms or other things? what causes it, the eggs we put in? the white ones or the black ones?” P2C.* Participant expressing confusion about the nature of cancer and its causes.

*“ Cancer is not a disease. It’s a demon, it’s a demon… We tried to find out who in the family could have this. We sat down with my mother to find out. She says she’s never heard of it, never seen it, not an aunt, not a grandmother or anything like that. She doesn’t know where it comes from... I had a husband who had this...maybe it’s...is it contagious?” P6S.*

*“I don’t know anything about the origin of the illness, the depths or stages of the illness and everything else...bad luck, I tell myself it’s bad luck...maybe it’s a spirit this illness...”P3S.*

*“Does God send diseases? There are only sorcerers, enemies... He’s an enemy, when he doesn’t want to see you, he puts a curse on you, that’s all...” P8S.*

#### Psychological repercussions of cancer- and cancer treatment-related physical disability.

The burden of illness and treatment intensity frequently resulted in patients relying heavily on family members for their daily activities, which some patients perceived to be infantilizing and devaluing:

*“I do nothing anymore, almost nothing...” P7S.*

On the other hand, physical limitations, engendered feelings of anger and frustration in patients:

*“Even when I wipe the table, I am out of breath afterward... it really annoys me...” P7S.*

Additionally, the physical limitations and side effects of treatment contributed to a profound sense of loss of self-identity:

*“I now walk slowly because it hurts me if I walk fast. I am now like a baby… It’s no longer the same life; it’s no longer the same person...” P4S.*

#### Psychological repercussions arising from the perceived level of social relations and support received after cancer disclosure to patients’ relatives.

For numerous patients, disclosing their diagnosis to adult children or close relatives emerged as an important source of comfort and resilience. Patients articulated sentiments of encouragement, liberation, and enhanced solidarity, as exemplified by these patients’ reflections:

*“ I spoke to my son, I saw his reaction... it calmed me down, yes it did...” P1S.*

*“I immediately called my daughter...I called my boss at work...”. P1C*

*“I called my little sister. I went to find her at her place of work and she immediately took me to another doctor...”. P7S.*

Conversely, some patients expressed apprehension about sharing their diagnosis with their children, fearing that it might impose an undue emotional burden of them. This process necessitated careful consideration:

*“My children are too young to understand... one is 16 years old...” P6S.*

Married women and individuals in committed relationships frequently confided in their spouses, occasionally extending this disclosure to other family members or colleagues.

*“I told my husband, and he collapsed... he was crying his eyes out...” P2S*

*“My little brother saw death...the last one was quite strong and my eldest daughter denied it, she asked me to see other doctors, to have other tests...You can’t share your experience with everyone, not everyone understands you... you have to hide it and that makes you sad. When you tell people, it makes them sad too... “ P4S*

*In some instances, the announcement of a diagnosis led to feelings of isolation within families: “My sisters do not call me to get news or to know what stage I am at...” P1C.*

*“My brothers and sisters came to visit me... Since they left, they have not come back...” P2C.*

The disease alters mood, emotions, judgement and quality of life.

*“When I have no energy left, it affects my emotions......sometimes I wake up angry and upset...every time I know I’m due to have a chemo session, I’m already angry...” P4S*

*“Since I’ve had cancer, I’ve had the impression that other people look at me and say “you’ve had it”. I’m not blaming them, but that’s how I feel. People don’t come up to me anymore, I always go up to them to avoid being alone...” P6S*

Sharing the diagnosis within the professional sphere often elicited sadness and led to reduced work activity due to treatment side effects, with some perceiving a distancing behavior from friends and colleagues, likely stemming from anxieties surrounding the illness:

*“They changed their behavior, and they changed their gaze. It is as if you are going to die...” P6S.*

*“Often my colleagues... some to whom I explained this would start crying...” P3S.*

*“I no longer work... I am forced to stop...” P4S*

*Mechanisms for coping with the psychological repercussions of cancer disclosure*

Spirituality and mystical-religious representations were observed as prominent coping mechanisms. These belief systems were perceived as primary strategies for managing suffering. For instance, the conceptualization of illness as a malevolent entity illustrates this the importance attributed to faith by patients as a coping mechanism for the psychological repercussions arising from breast or cervical cancer disclosure. Spiritual engagement plays a central role in patient resilience, with many individuals deriving solace from prayer and the study of sacred texts, further emphasizing the perceived significance of faith in their healing process. Furthermore, patients frequently expressed conviction in divine intervention. The role of faith and divine intervention in the healing process, as perceived by patients, is vividly expressed in their verbatim. One patient, for instance, conveyed with certainty: *“My faith has allowed me to experience certain things... therefore, I cannot doubt God today because I am ill, no... I cannot doubt Him. I have lived so many things, so many things, and I have felt His hand in my life, so I remain ever confident, I remain ever confident...” P1S.*

However, the utilization of faith-based coping strategies is not without inherent challenges. As one patient articulated, the complexity of emotional responses in the face of illness and mortality remains significant. This is echoed in the sentiments of another participant who stated: *“Even when you have faith... I believe in God but... it’s not easy.”P4S.* This brief but poignant statement highlights that even for individuals who hold strong religious beliefs, the emotional and existential burden of cancer is not easily alleviated by faith alone.”

Resilience also manifested as a salient adaptive response, wherein individuals demonstrated the capacity to mobilize courage despite experiencing pain and anguish. Conversely, isolation and the pursuit of solitude were also employed as coping mechanisms. Social isolation represented a response to social anxiety. Because patients feared judgment or rejection, they withdrew to mitigate anxiety-provoking social events. One patient described this period of withdrawal as follows*: “...I no longer wanted to see people, I shut myself away... the phone was far away so that people wouldn’t disturb me...” P4S.*

Despite the myriad challenges, some patients described experiencing personal growth stemming from their experience with illness. They reported a newfound appreciation for life and a shift towards living in the present moment: *“This illness has taught me... today every day counts, every minute counts for me...” (P4S).*

Social support from family and friends emerged as a critical factor for coping with the emotional and physical burdens associated with the disease.

Table 4 shows some of the coping strategies adopted by patients.

**Table 4:** Patients’ defense and coping mechanisms.

Theme	Sub-theme	Definition	Verbatim	Patient’s identity
**Defense mechanisms**	Defense mechanisms, initially conceptualized by Anna Freud (1936), are understood as unconscious psychological processes through which the ego protects itself from internal conflicts arising from instinctual drives and their associated affects. These defensive operations, whether manifested as maneuvers or measures, are intrinsically involuntary in their activation [35]			
	**Isolation**	Isolation, as a defense mechanism, is characterized by a dissociation between the cognitive representations of an experience and the affects initially associated with it. The individual retains conscious awareness of the factual or ideational elements but loses contact with the original emotional valence linked to these representations [36]	*“...I no longer wanted to see people, I shut myself away... the telephone was far away so that people wouldn’t disturb me...”* *“I needed to go to the beach. I needed to be alone with myself, not with those who wanted to tell you what to do. I needed to hear myself and not what people were going to say to me... when I got home, I stayed in my room for a long time, I was reflecting...”* *“When I got home, I locked myself in my room, I wasn’t eating anymore...”*	P4S P4S P9S
	**Denial**	The individual employs a situational management strategy characterized by the denial of certain aspects of the reality inherent to their experience, despite their obviousness to an external observer. This psychological distancing is achieved through the selective concealment of specific constituent elements of the problem [35].	*“I took it as if it were nothing, I didn’t let it sink in...”* *“I know I am sick, but it’s as if I had malaria...”* *“I know I went through a sort of denial for a while...I was told that it was a tumor. When he told me, I laughed because I said, ‘Doctor, are you joking?’ At first, I refused; I thought he was joking...”*	P8S P10S P4S
	**Repression**	Repression is a psychological response to conflict and stress, characterized by the deliberate avoidance of thoughts, desires, feelings, or aversive experiences. These disturbing contents are relegated to the preconscious, remaining potentially accessible. Repression can be conceptualized as a reversible and adaptive form of forgetting [37]	*“In the silence. In the silence. I try to...I try to think of something else; I try to laugh with the others to forget but...it’s just in the* *the moment. When you find yourself in your room alone, it’s pitch black”.*	P6S
**Coping mechanisms**	*Coping is conceptualized as the totality of cognitive and behavioral efforts that individuals employ to manage the internal or external demands of a situation appraised as stressful and exceeding their available psychological resources* [38].			
	**Meaning-centred coping**	Meaning-focused coping, distinct from problem- and emotion-focused strategies centers on seeking social support (help, encouragement, sympathy). Patients express significant gratitude towards their social network (family, friends, caregivers) perceived as essential support in navigating the illness [39].	*“I read the Bible and meditations, that’s what keeps me going...the Bible and prayers have intensified since I’ve been ill, it helps me to keep going...”* *“I’m a Catholic Christian, and thanks to Psalms 48, 42 and 50, I feel good... “* *“I know that God works miracles...* *“There are miracles, God can work miracles and then you’re healed. There’s only the Lord Jesus. In all things, it’s God...”* *“God allows it to strengthen my faith. I told my husband and my children, all you have to do is pray... that’s all I ask of you. I’ll be fine with God’s grace.... “*	P1S P2C P3S P2S
	**Emotion-focused coping**	Emotion-focused coping strategies aim to modulate the intensity of negative affects through various approaches: avoidance, minimization, distancing, selective attention, positive comparisons, and positive reappraisal. Patients may use emotion regulation techniques (relaxation, meditation, group support) or adopt avoidance behaviors (denial) or positive reinterpretation (benefit-finding) [39]	*“I don’t ask God why you’ve done this to me... I tell him if you’ve done this to me, it’s because... you’ve given me the strength I need to... the strength he’s put in me, I’m going to put it to the end because I want to live...”.* *“I pray to the Lord, he’s the one who has the breath of life. If God says I’m leaving first, I’m leaving. If God hasn’t decided yet, I’ll be there despite the illness...”.* *“Even when you have faith... I believe in God but... it’s not easy...”*	*P4S* *P8S* *P4S*

### Impact of the healthcare professional’s attitude on patients’ mental health after cancer disclosure

11 of the 13 participants had their cancer discovered fortuitously after undergoing clinical and paraclinical assessments for non-specific symptoms:

*“I noticed that my periods were taking longer than usual...”. P3C,*

*“I was already post-menopausal, I saw blood...a bit of blood and after 2 days, 3 days, it stops. And I had a big belly...”. P2C,*

*“...I didn’t have a lump or anything...but every time I washed, I still felt a bit of pain, and the lymph node under my armpit got bigger...” P2S.*

Others ignored the symptoms and waited for a few more days, months or even years before going to hospital:

*“I felt a weight pressing down on me, but I neglected it. I thought that with time, it would go away...but I’m a health worker...” P10S*

*“Doctor told me to go for a breast ultrasound...I didn’t go...”. P8S.*

Most patients (10/13) had their diagnosis being made by a gynecologist, while two saw a medical oncologist and the last one a general nurse. Nine patients had their diagnosis being disclosed while they were alone, in consultation with the doctor. Four patients said they had been accompanied, including one by her son, the second one by her sisters-in-law, the third one by her parents and the father of her children, and the fourth one by her sister and one of her close friends. The quality of the announcement was judged to be ‘poor’ or ‘too brutal’ in some cases, to the extent that some of them said they had no recollection of what the doctor had said:

*“it was really brutal the way it was done…” P3C*

*“I didn’t like the announcement…it wasn’t done well…” P5S*

*“I was too desperate, I spent my time crying, my mind was elsewhere, I didn’t memorize anything the doctor had said.” P9S.*

Others reported feeling supported and informed: *“The doctor did it calmly; he was composed. I even asked him if he could treat me, and he said yes. I asked if he could operate on me, and he said yes. So, I thought, if the doctors could treat me...” P4S*

Table 5 summarizes the specific needs expressed by each patient and the focus group regarding the qualities, they deemed to be important for ensuring a positive patient-caregiver relationship, and which they would have liked to find in the individual disclosing the diagnosis to them. We also took care to note, for each patient, the individuals who disclosed the diagnosis to them.

**Table 5:** Interview-derived patients’ needs with regard to attending caregivers’ attitudes during cancer disclosure consultations.

Patient’s identity	Healthcare provider	Interpersonal skills	Professional know-how
P1S	Oncologist	Open to dialogue, Respect, Trust/Assurance	Knowledgeable
P2S	Oncologist	Open to dialogue, Frankness/Truth, Respect, Compassion/Tolerance, Empathy, Trust/Assurance	Knowledgeable
P3S	Gynecologist	Open to dialogue, Frankness/Truth, Respect, Compassion/Tolerance, Trust/Assurance	Knowledgeable
P4S	Gynecologist	Open to dialogue, Respect, Compassion/Tolerance, Empathy, Approachable/welcoming	Knowledgeable
P5S	Gynecologist	Open to dialogue, Frankness/Truth, Respect, Empathy, Trust/Assurance, Approachable/welcoming	Knowledgeable
P6S	Gynecologist	Open to dialogue, Frankness/Truth, Respect, Compassion/Tolerance, Empathy, Trust/Assurance	
P7S	Gynecologist	Open to dialogue, Frankness/Truth, Trust/Assurance	Knowledgeable
P8S	Gynecologist	Open to dialogue, Frankness/Truth, Respect, Compassion/Tolerance, Empathy, Trust/Assurance	Knowledgeable
P9S	Gynecologist	Open to dialogue, Frankness/Truth, Respect, Compassion/Tolerance, Empathy, Approachable/welcoming	
P10S	Gynecologist	Open to dialogue, Frankness/Truth, Trust/Assurance	
P1C	General nurse	Open to dialogue, Frankness/Truth, Respect, Compassion/Tolerance, Empathy, Approachable/welcoming	
P2C	Gynecologist	Open to dialogue, Frankness/Truth, Respect, Compassion/Tolerance, Empathy, Trust/Assurance	
P3C	Gynecologist	Open to dialogue, Frankness/Truth, Compassion/Tolerance, Empathy, Trust/Assurance, Approachable/welcoming	Knowledgeable

Communication, trust, compassion, skills, and availability active listening proved to be essential for establishing a good patient-caregiver relationship, as well as the person responsible for disclosing the diagnosis.

Patients expressed the need for their caregiver to carefully choose their words while announcing a cancer diagnosis, with the words “cancer” and “tumor” deemed to be unacceptable due to their very painful and morbid connotations:

*“The only word I don’t like is “tumor” because the last time I was watching... I was in my head and then I thought to myself, but ‘tumor’ is really not good because it starts with ‘tu’ and ends with ‘meurs’ (you die), so ‘tumor’ is not a good word. I no longer say ‘tumor’ or ‘cancer,’ but ‘neoplasm,’ and that... that sounds better, it’s softer”. P4S*

*“Tumor means ‘you die, cancer, no. These are strong words.” P1C.*

Patients identified several critical attributes of healthcare providers during the disclosure of diagnoses. Foremost among these is clear and empathetic communication, which ensures that medical information is conveyed in an accessible and reassuring manner. Trust, compassion, and respect were also highlighted as essential qualities that mitigate emotional distress. The importance of availability and active listening was underscored, as these allow patients to express their concerns freely. Furthermore, sincerity and transparency were valued, promoting an honest yet sensitive approach. Professional competence and psychological attunement further bolster patient confidence in the disclosure process. The manner in which caregivers communicate during diagnosis can significantly influence patients’ emotional responses and engagement with treatment. A structured and supportive approach may facilitate more adaptive coping mechanisms and foster stronger therapeutic relationships.

## Discussion

This mixed-methods study provides critical insights into the psychological repercussions experienced by Gabonese women following the disclosure of a breast or cervical cancer diagnosis. The findings demonstrate substantial emotional distress, depression severity scales ranging from minimal to moderately severe on PHQ-9, as well as multiple other negative psychological repercussions potentially arising from cancer/cancer treatment-related physical disability and self-perceived levels of social support from their relatives. Accordingly, patients reported having negative perceptions and representations about cancer, and exhibited various coping and defense mechanisms. Moreover, patients expressed concerns about the use of the words “cancer” and “tumor” during the disclosure consultation and a need for knowledgeable health professionals with interpersonal skills for the announcement of a cancer diagnosis. This assessment aligns with the international literature, which emphasizes a the significant psychological burden accompanying the disclosure of cancer diagnoses, by a constellation of anxiety, depression, fear of death, and perceptions of altered body image [14,40–46].

The qualitative data underscore the traumatic impact of diagnosis disclosure, highlighting the necessity for improved communicative practices among healthcare professionals [47]. Some patients described the health professionals conducting the disclosure as being brutal or insufficiently supportive, and those undesirable attitudes exacerbated their emotional distress and affected their ability to recall critical information.

Our study demonstrated that spirituality functioned as a significant coping mechanism for numerous participants, aligning with research in similar contexts where religious beliefs are pivotal in managing psychological distress associated with illness [48,49]. This reliance suggests the potential benefit of integrating psychosocial and spiritual support within cancer care pathways in Gabon. Additionally, the importance of familial and social support for coping and resilience was evident in our data, corroborating existing literature that emphasizes the positive impact of robust support networks in navigating cancer [50,51]. Conversely, instances of isolation or distancing from family and friends were associated with increased vulnerability, indicating an area where healthcare systems need to enhance support for psychosocial well-being among cancer patients. Finally, the identified challenges in patient-provider communication underscore a critical need for targeted training programs aimed at enhancing healthcare professionals’ skills in empathetic communication, active listening, and supportive counseling, as perceived by the participants in our study.

Previous studies had similar findings, thus emphasizing the importance of empathetic communication and information clarity during cancer diagnosis disclosure to mitigate immediate psychological distress and promote patient resilience [46,52–54].

### Strengths and limitations of the study

This pilot exploratory study addresses a significant research gap in Gabon by investigating psychological impacts following breast and cervical cancer diagnosis disclosures—an area seldom explored in this specific context. By highlighting these psychological dimensions, our research contributes foundational insights that can inform future comprehensive investigations and holistic management approaches for cancer care in Gabon.

Nevertheless, this study presents several limitations. It was conducted in a single hospital setting with limited sample size. These drawbacks limit the generalizability of findings from this study even at the national level. It is likely that a recall bias was introduced due to the lag time between cancer diagnosis and disclosure data collection. Indeed, this may have influenced the accuracy of patient responses. The social desirability bias could also have resulted in under-reporting of negative emotional experiences. Furthermore, the cross-sectional data collection and analysis precluded the assessment of the long-term psychological trajectories following cancer diagnosis. Although the research identified the needs of patients regarding the attitudes of attendant health professionals during the disclosure consultation, it did not explore the specific psychological interventions that are needed to improve the cancer disclosure process and the psychological well-being of patients throughout their cancer journey,

Considering these limitations, future research should prioritize larger, longitudinal and multicenter studies with diverse populations, within and across borders with longitudinal to understand the long-term psychological of breast or uterine cervical cancer disclosure, and consequently evaluate the most suitable targeted psychological interventions. In the meantime, contextually tailored recommendations for clinical practice and policy development are necessary in Gabon, taking into account the unique socio-cultural aspects of the country. This includes focusing on therapeutic communication training for healthcare providers, emphasizing active listening and empathy, to mitigate psychological distress and enhance resilience.

## Conclusion

Cancer disclosure in Gabonese women with breast and cervical cancers resulted in important psychological distress, including anxiety, depression, fear of death, and negative body image, exacerbated by perceived inadequate empathetic communication during diagnosis. Future research should prioritize the development of culturally tailored psychological interventions to address the specific needs of these patients.

## Supporting information

S1 Appendix COREQ checklist. (DOCX)

S2 Appendix STROBE checklist. (DOCX)

S3 Appendix PHQ-9 questionnaire. (DOCX)
