# Supplementary material for: Psychological repercussions of breast or uterine cervical cancer disclosure to women in Gabon
Source: PLoS One. 2025 Jun 20;20(6):e0326378. doi: 10.1371/journal.pone.0326378 (PMC12180733; doi:10.1371/journal.pone.0326378)
Supplement: S3 Appendix — (DOCX) [file pone.0326378.s003.docx]

S3 Appendix. PHQ-9 questionnaire
